# Supplementary material for: Nestin-dependent mitochondria-ER contacts define stem Leydig cell differentiation to attenuate male reproductive ageing
Source: Nat Commun. 2022 Jul 11;13:4020. doi: 10.1038/s41467-022-31755-w (PMC9276759; doi:10.1038/s41467-022-31755-w)
Supplement: Supplementary file 1 — Supplementary Information [file 41467_2022_31755_MOESM1_ESM.pdf]

Supplemental Information

Nestin-dependent mitochondria-ER contacts define stem Leydig cell differentiation to attenuate male reproductive ageing

Yao et al.

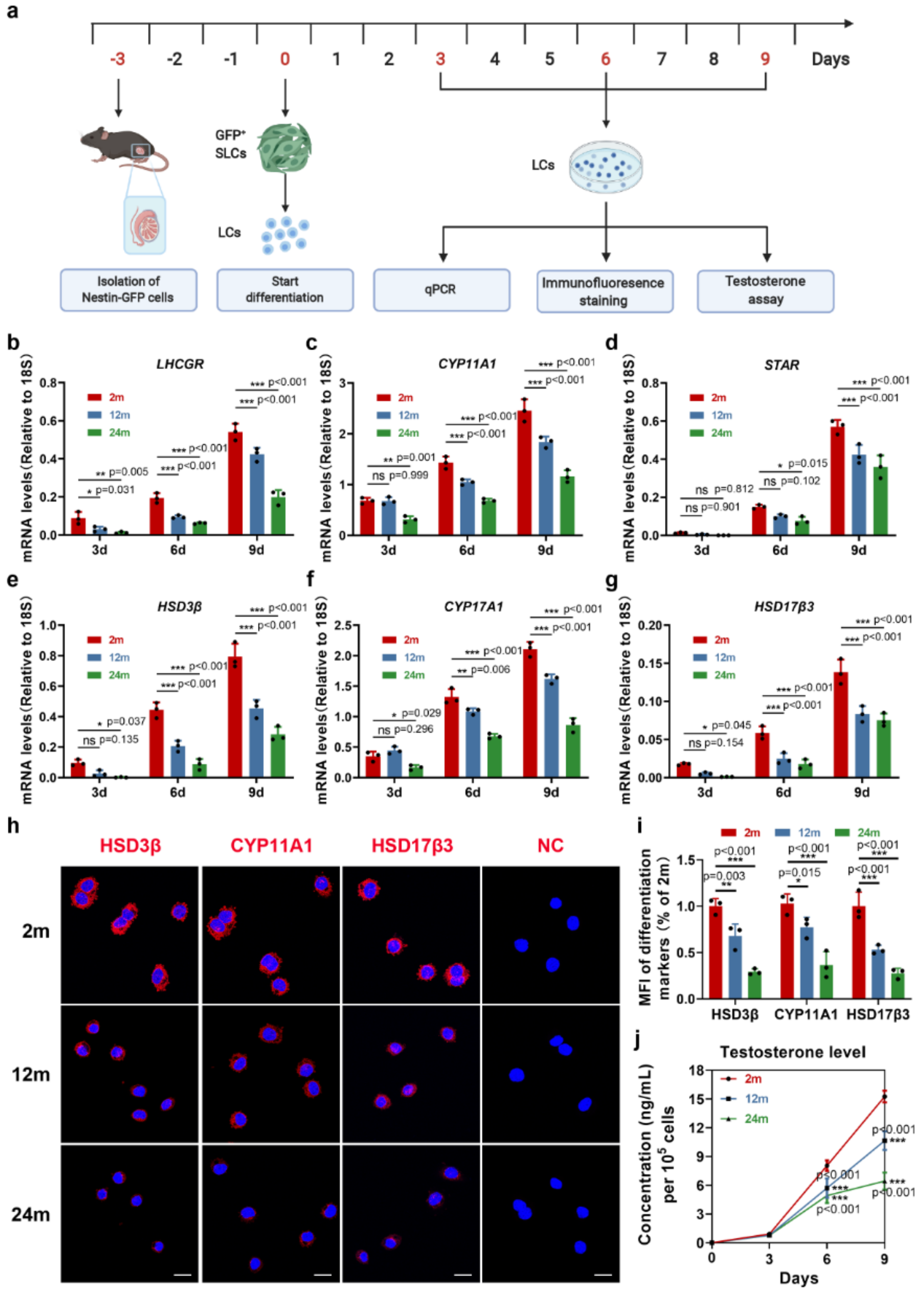

Supplementary Fig. 1 | The effect of ageing on induced differentiation of SLCs into LCs in vitro.

(a) Schematic of the experimental procedure for isolating primary Nestin-GFP+ SLCs and inducing differentiation into LCs. Created with BioRender.com.

(b-g) qPCR analysis of relative mRNA expression of testosterone production related genes at different time points (day 3, day 6, day 9) during induced differentiation of primary Nestin-GFP+ SLCs from different age groups. (n = 3 biological repeats for each group; All data are mean ± SD; Two-way ANOVA).

(h) Representative immunostaining pictures of LCs induced from primary Nestin-GFP+ SLCs from different age groups at day 9. LCs are identified as HSD3β+/CYP11A1+/HSD17β3+ cells. Scale bar, 20 μm.

(i) Quantification of the mean fluorescent intensity in (h) (n = 3 biological repeats for each group; All data are mean ± SD; Multiple t tests).

(j) Quantification of testosterone level in the supernatants of medium during induced differentiation of primary Nestin-GFP+ SLCs from different age groups (n = 3 biological repeats for each group; All data are mean ± SD; Two-way ANOVA).

Two-sided comparison; Error bars represent SDs. $*p < 0.05$, $**p < 0.01$, $***p < 0.001$.

Source data are provided as a Source Data file.

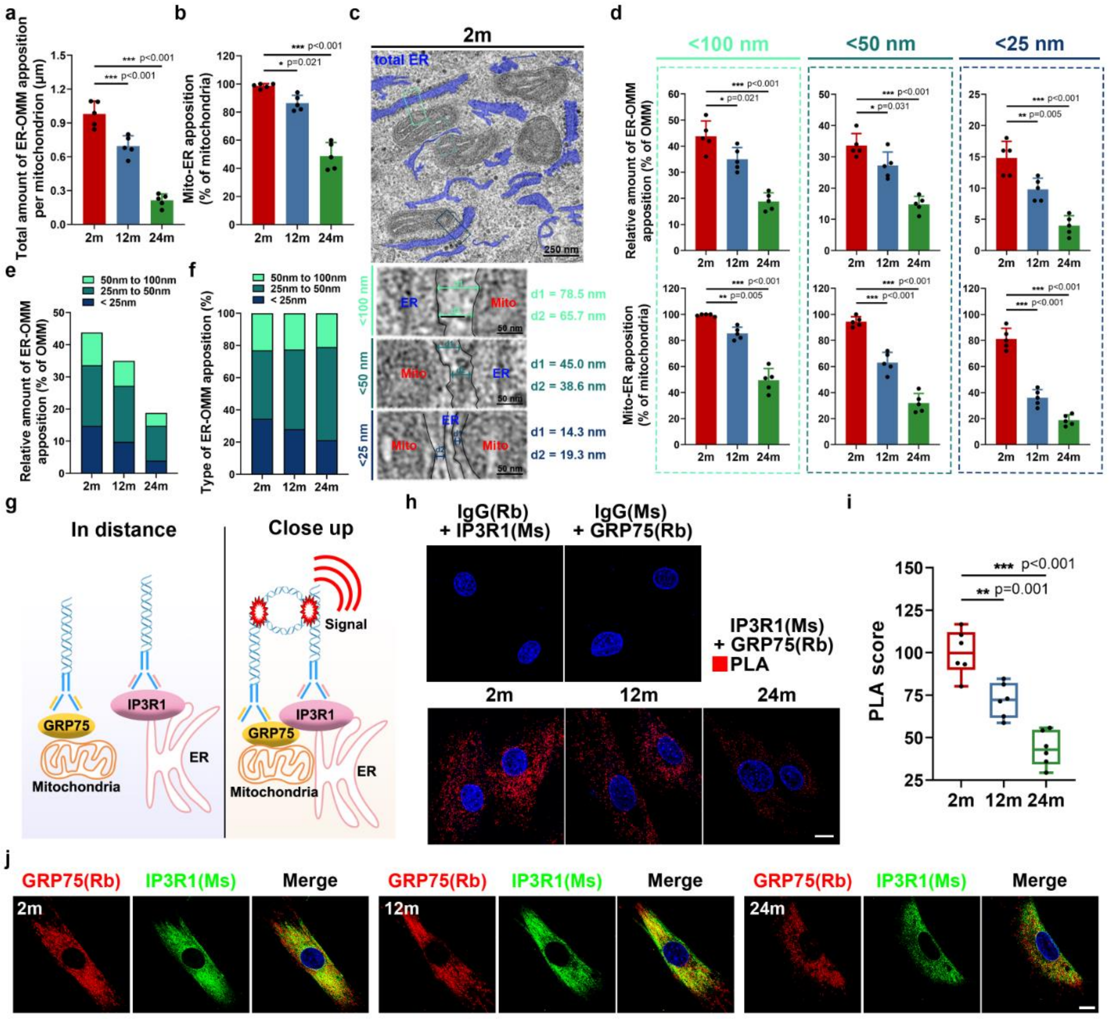

Supplementary Fig. 2 | MERCs of all intermembrane distances was reduced during SLC ageing.

(a) Quantification of the extent of MERCs (<100 nm) from **Figure. 2e** in primary Nestin-GFP+ SLCs from different age groups. The total apposition length refers to the interaction length of ER tubules within 100 nm of the OMM covered by contacts per mitochondria (n =26 to 48 mitochondria in 5 fields per condition; All data are mean ± SD; One-way ANOVA)

(b) Quantification of percentage of mitochondria in contact with the ER (<100 nm) in total mitochondria per field of view from **Figure. 2e** in primary Nestin-

GFP+ SLCs from different age groups. (n =26 to 48 mitochondria in 5 fields per condition; All data are mean ± SD; One-way ANOVA)

(c) Representative TEM image of primary Nestin-GFP+ SLCs from 2 months age group highlighting ER-OMM distances of less than 100, 50 and 25 nm. Scale bar, 250 nm for original pictures and 50 nm for enlarged pictures.

(d) Quantification of the relative amount of OMM in contact with the ER (top) and percentage of mitochondria in contact with the ER in total mitochondria per field of view (bottom) for each of the three ER-OMM distance categories from (c), for cells from different age groups. (n =20 to 42 mitochondria in 5 fields per condition. All data are mean ± SD; One-way ANOVA).

(e,f) Distribution of ER-OMM contact in primary Nestin-GFP+ SLCs from different age groups, displayed as the percentage of OMM corresponding to each intermembrane distance (e) or as a percentage of all ER-OMM contacts (f).

(g) Schematic of the experimental mechanism of proximity-ligation-assay (PLA) detecting the proximity between mitochondria and ER.

(h) Representative PLA staining pictures of colocalization between GRP75 and IP3R1 in primary Nestin-GFP+ SLCs from different age groups. Scale bar, 10 μm.

(i) Analysis of PLA score in (j) (n = 6 biological repeats for each group; Box plots indicate the median [center line inside the box], lower and upper

quartiles [bounds of box], smallest and largest values [whiskers], One-way ANOVA).

(j) Representative control image of antibodies for immunofluorescent staining used in PLA (h). Scale bar, 10 μm.Error bars represent SDs. $*p < 0.05$, $**p < 0.01$, $***p < 0.001$.

Source data are provided as a Source Data file.

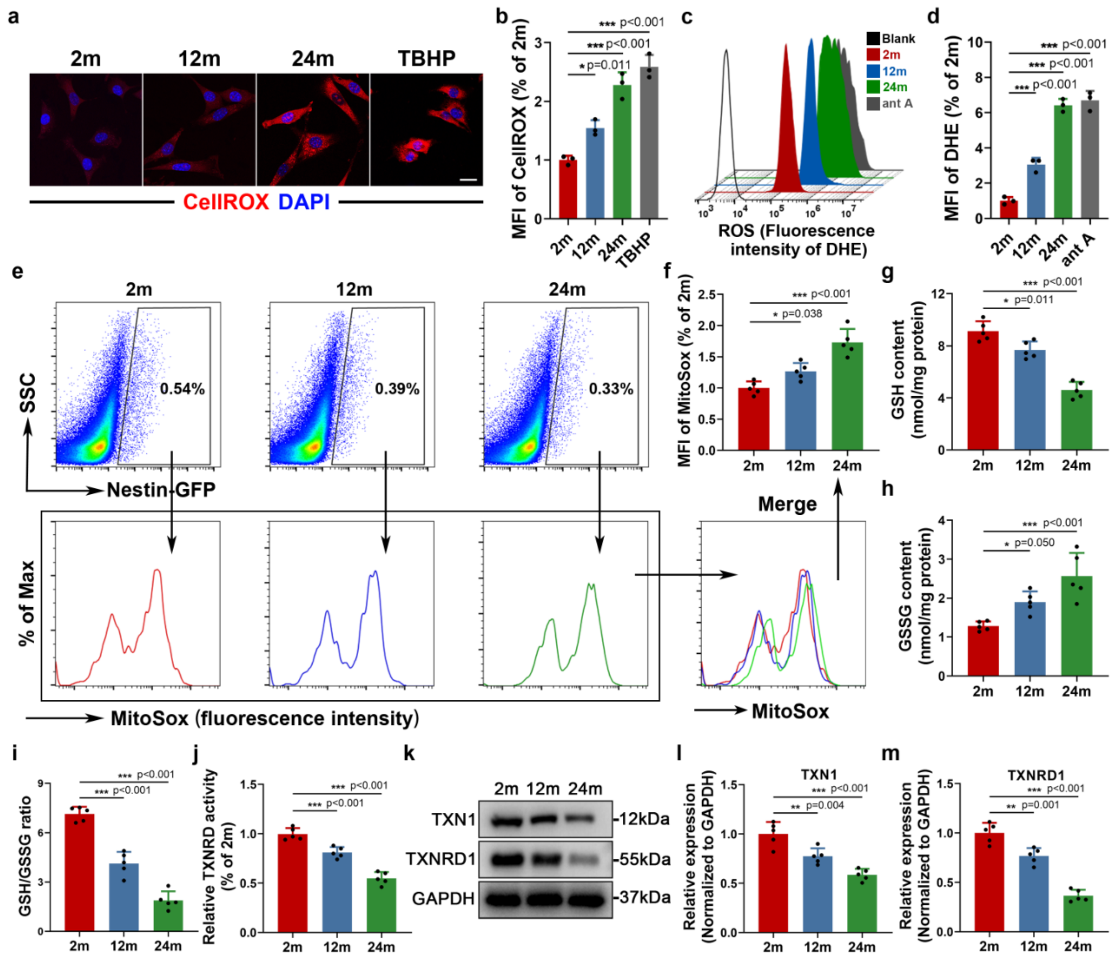

Supplementary Fig. 3| Increased intracellular ROS levels and reduced antioxidation ability during SLC ageing.

(a) Representative immunostaining pictures of intracellular ROS stained with CellRox in primary Nestin-GFP+ SLCs from different age groups in vitro. Scale bar, 20 μm. 200 μM TBHP was the positive control added to the 2m group.

(b) Quantitative analysis of mean fluorescence intensity of CellRox in (a). (n = 3 biological repeats for each group; All data are mean ± SD; One-way ANOVA).

(c) Flow cytometry of intracellular ROS level stained with DHE of primary Nestin-GFP+ SLCs from different age groups in vitro. 100 μM antimycin was the positive control added to the 2m group.

(d) Quantification of mean fluorescence intensity of DHE in (c). (n = 3 biological repeats for each group; All data are mean ± SD; One-way ANOVA).

(e) Flow cytometry of mitochondrial ROS level stained with MitoSox of Nestin-GFP+ SLCs from different age groups in vivo.

(f) Quantification of mean fluorescence intensity of MitoSox in (e). (n = 5 biological repeats for each group; All data are mean ± SD; One-way ANOVA).

(g–j) the levels of GSH (g), GSSG (h), the ratios of GSH/GSSG (i) and the activities of TXNRD1 (j) were measured in the cytosol of Nestin-GFP+ SLCs from different age groups in vivo. (n = 5 biological repeats for each group; All data are mean ± SD; One-way ANOVA).

(k-m) Western Blot analysis and quantification of thioredoxin of primary Nestin-GFP+ SLCs from different age groups in vivo. (n = 5 biological repeats for each group; All data are mean ± SD; One-way ANOVA).

Error bars represent SDs. *p < 0.05, **p < 0.01, ***p < 0.001.

Uncropped western blots and source data are provided as a Source Data file.

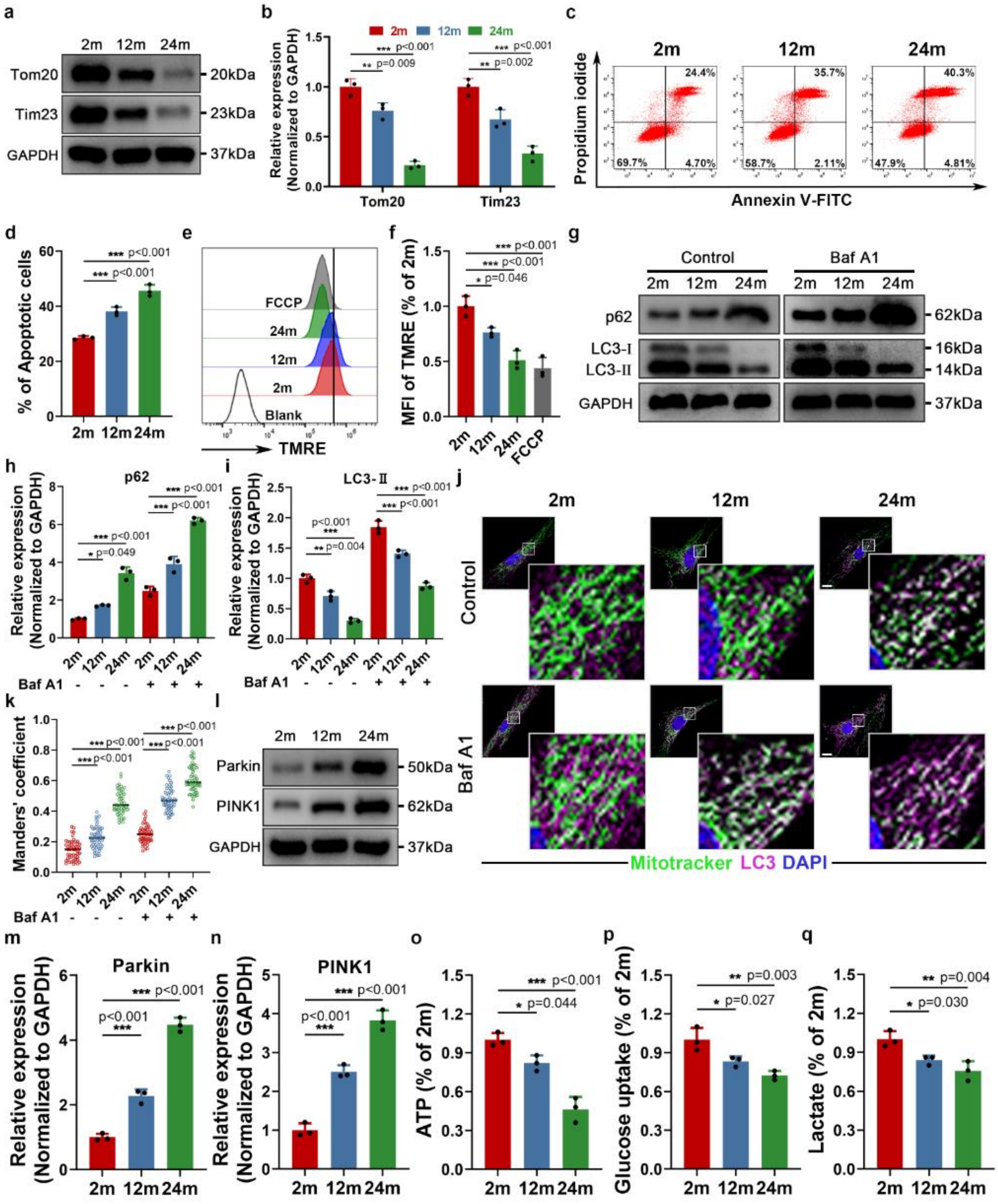

Supplementary Fig. 4 | Mitochondrial dysfunction and disordered redox homeostasis in aged SLCs.

(a-b) Western Blot analysis and quantification of mitochondrial proteins of primary Nestin-GFP+ SLCs from different age groups in vitro. (n = 3 biological repeats for each group; All data are mean ± SD; Multiple t tests).

(c) Flow cytometry analysis of apoptosis of primary Nestin-GFP+ SLCs from different age groups in vitro, double-stained with Annexin V-FITC and propidium iodine (PI).

(d) Quantification of apoptosis ratio of total cells from (e). (n = 3 biological repeats for each group; All data are mean ± SD; One-way ANOVA).

(e) Flow cytometry analysis of mitochondrial membrane potential stained with TMRE of primary Nestin-GFP+ SLCs from different age groups in vitro. 20 μM FCCP was the positive control added to the 2m group.

(f) Quantification of mean fluorescence intensity of TMRE in (g). (n = 3 biological repeats for each group; All data are mean ± SD; One-way ANOVA).

(g-i) Western Blot analysis and quantification of autophagy related proteins in Control (Vehicle) and Bafilomycin A1 (Baf A1) treated primary Nestin-GFP+ SLCs from different age groups in vitro. (n = 3 biological repeats for each group; All data are mean ± SD; One-way ANOVA).

(j) Representative immunostaining pictures of mitophagy in Control (Vehicle) and Bafilomycin A1 (Baf A1) treated primary Nestin-GFP+ SLCs from different age groups in vitro. Mitochondria are stained with Mitotracker and autophagy is stained with LC3. Scale bar, 10 μm.

(k) Quantitative analysis of mitophagy by calculating Mander's coefficient in (l). (Manders' coefficient of mitochondria is shown for each condition, n = 48, 53 and 48 cells for 2m, 12m and 24m SLCs, respectively; All data are mean ± SD; One-way ANOVA).

(l-n) Western Blot analysis and quantification of mitophagy related proteins in primary Nestin-GFP+ SLCs from different age groups in vitro. ($n = 3$ biological repeats for each group; All data are mean ± SD; Multiple t tests).

(o) Quantitative analysis of mitochondrial ATP production in primary Nestin-GFP+ SLCs from different age groups in vitro ($n = 3$ biological repeats for each group; All data are mean ± SD; One-way ANOVA).

(p) Quantitative analysis of glucose uptake primary Nestin-GFP+ SLCs from different age groups in vitro ($n = 3$ biological repeats for each group; All data are mean ± SD; One-way ANOVA).

(q) Quantitative analysis of lactate production of primary Nestin-GFP+ SLCs from different age groups in vitro ($n = 3$ biological repeats for each group; All data are mean ± SD; One-way ANOVA).

Two-sided comparison; Error bars represent SDs. *$p < 0.05$, **$p < 0.01$, ***$p < 0.001$;

Uncropped western blots and source data are provided as a Source Data file.

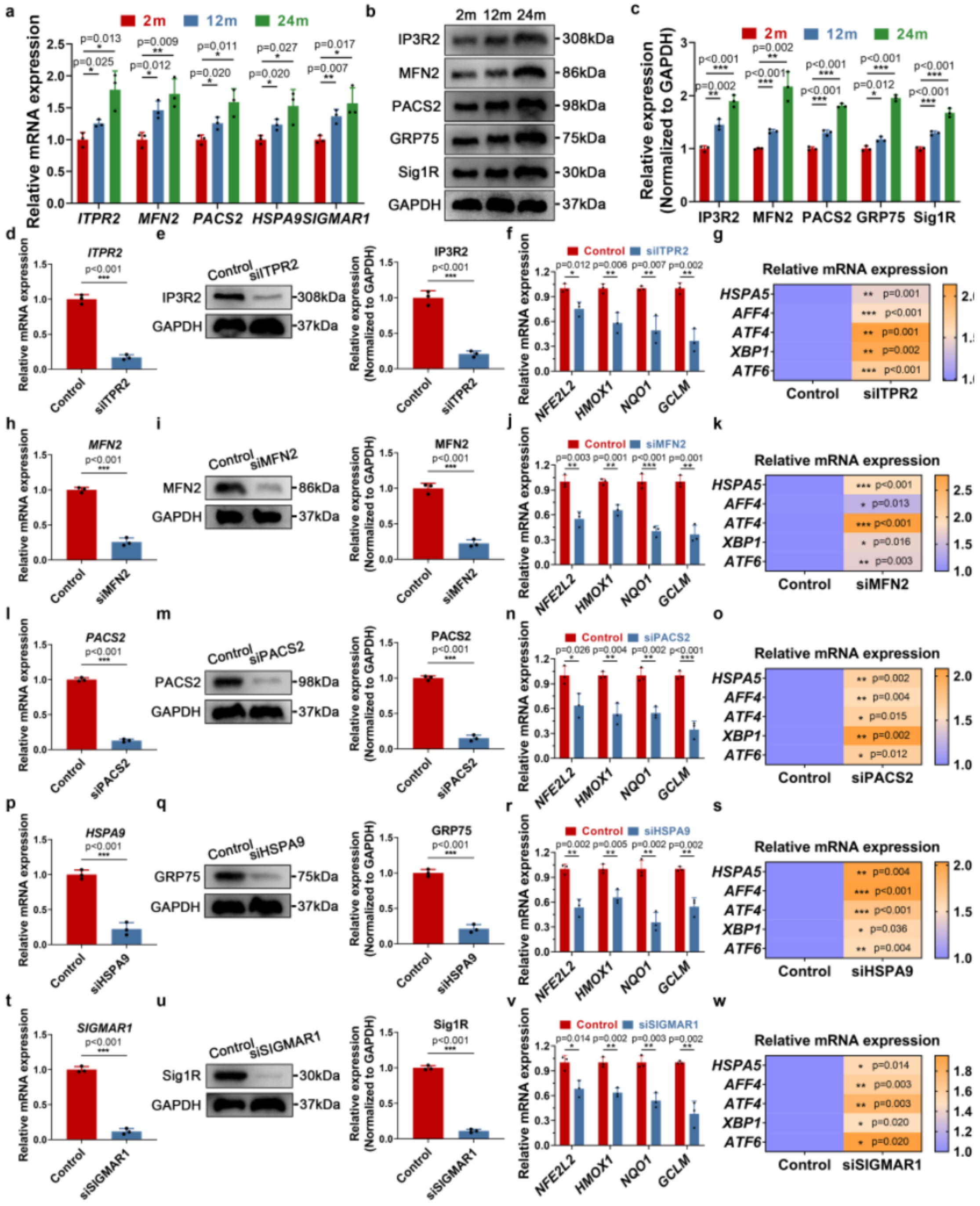

Supplementary Fig. 5 | The change of expression of MAMs resident proteins and their effects on REDOX and ER stress during SLC ageing.

(a) qPCR analysis of relative mRNA expression of MAMs resident proteins in primary Nestin-GFP+ SLCs from different age groups in vitro. (n = 3 biological repeats for each group; All data are mean ± SD; Multiple t tests).

(b-c) Western Blot analysis and quantification of MAMs resident proteins expression in primary Nestin-GFP+ SLCs from different age groups in vitro. (n = 3 biological repeats for each group; All data are mean ± SD; Multiple t tests).

(d-g) qPCR analysis of relative mRNA expression of IP3R2 (d), Western Blot analysis and quantification of IP3R2 protein expression (e) and qPCR analysis of relative mRNA expression of anti-oxidative (f) and ER stress (g) related genes of IP3R2-knockdown primary SLCs from 2 months old mice. (n = 3 biological repeats for each group; All data are mean ± SD; Multiple t tests).

(h-k) qPCR analysis of relative mRNA expression of MFN2 (h), Western Blot analysis and quantification of MFN2 protein expression (i) and qPCR analysis of relative mRNA expression of anti-oxidative (j) and ER stress (k) related genes of MFN2-knockdown primary SLCs from 2 months old mice. (n = 3 biological repeats for each group; All data are mean ± SD; h-i, unpaired t test; j-k, multiple t tests).

(l-o) qPCR analysis of relative mRNA expression of PACS2 (l), Western Blot analysis and quantification of PACS2 protein expression (m) and qPCR analysis of relative mRNA expression of anti-oxidative (n) and ER stress (o) related genes of PACS2-knockdown primary SLCs from 2 months old mice. (n = 3 biological repeats for each group; All data are mean ± SD; l-m, unpaired t test; n-o, multiple t tests).

(p-s) qPCR analysis of relative mRNA expression of GRP75 (p), Western Blot analysis and quantification of GRP75 protein expression (q) and qPCR

analysis of relative mRNA expression of anti-oxidative (r) and ER stress (s) related genes of GRP75-knockdown primary SLCs from 2 months old mice. (n = 3 biological repeats for each group; All data are mean ± SD; p-q, unpaired t test; r-s, multiple t tests).

(t-w) qPCR analysis of relative mRNA expression of Sig1R (t), Western Blot analysis and quantification of Sig1R protein expression (u) and qPCR analysis of relative mRNA expression of anti-oxidative (v) and ER stress (w) related genes of Sig1R -knockdown primary SLCs from 2 months old mice. (n = 3 biological repeats for each group; All data are mean ± SD; t-u, unpaired t test; v-w, multiple t tests).

Two-sided comparison; Error bars represent SDs. *p < 0.05, **p < 0.01, ***p < 0.001.

Uncropped western blots and source data are provided as a Source Data file.

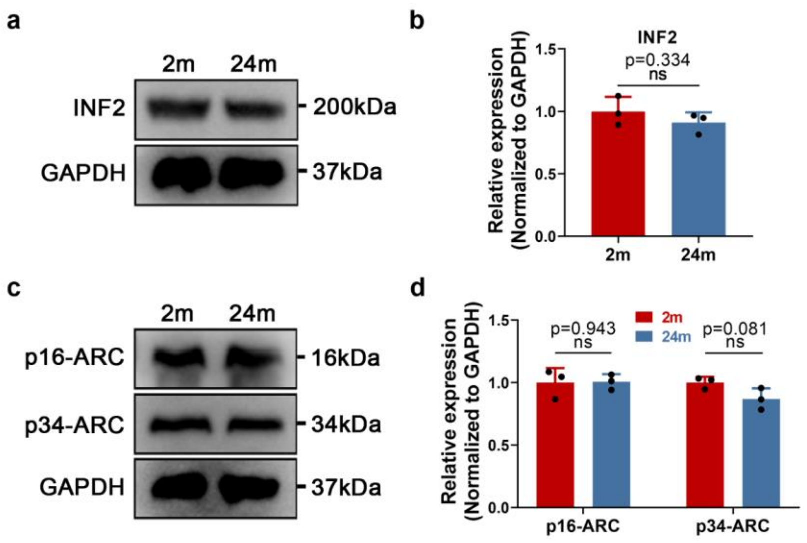

Supplementary Fig. 6 | INF2 and actin branching factors were stable between young and aged SLCs.

(a-b) Western Blot analysis and quantification of INF2 expression in primary SLCs from 2 months and 24 months old mice; (n = 3 biological repeats for each group; All data are mean ± SD; Unpaired t test).

(c-d) Western Blot analysis and quantification of actin branching factors expression in primary SLCs from 2 months and 24 months old mice; (n = 3 biological repeats for each group; All data are mean ± SD; Multiple t test).

Two-sided comparison; Error bars represent SDs. ns, $p > 0.05$.

Uncropped western blots and source data are provided as a Source Data file.

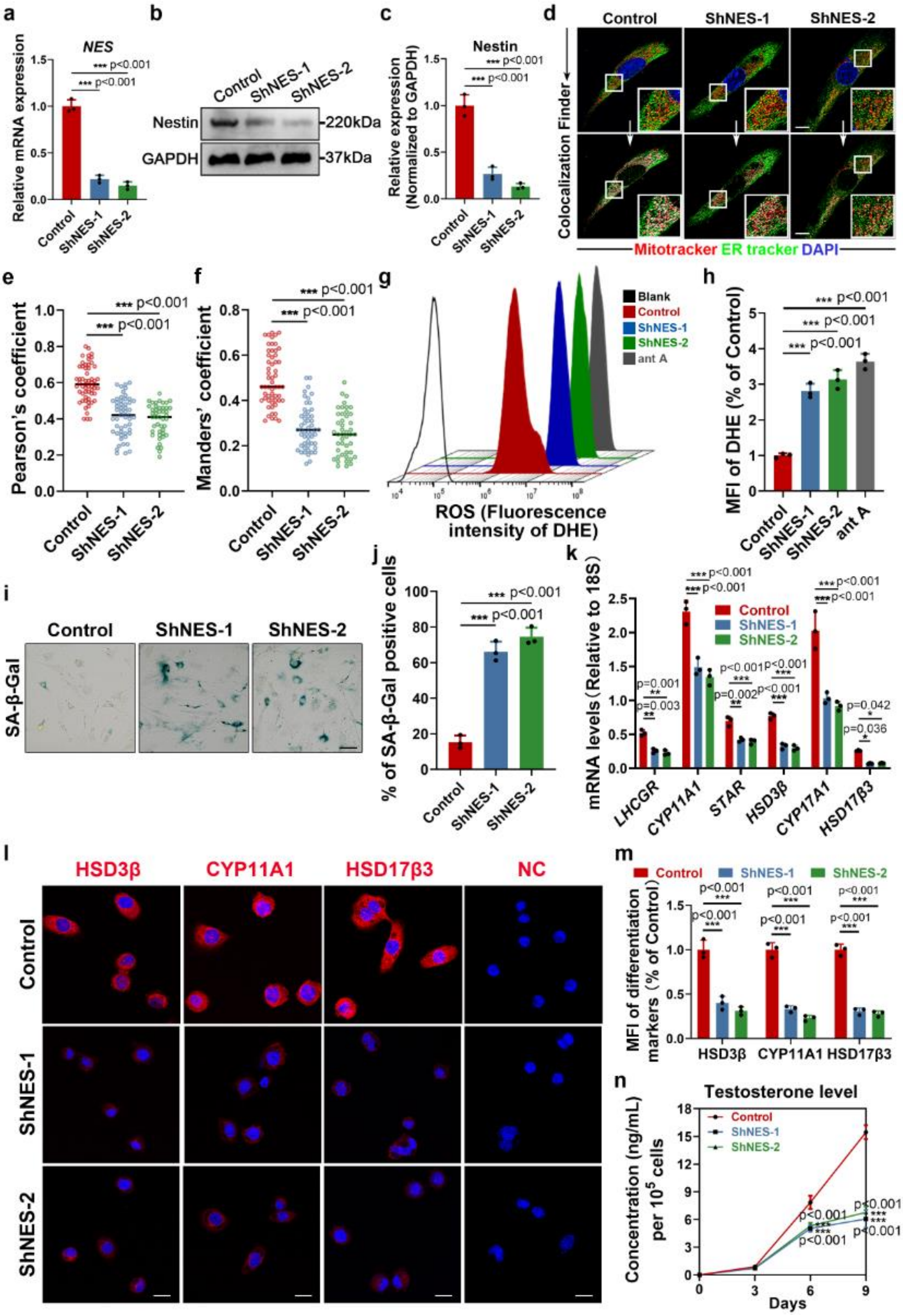

Supplementary Fig. 7 | Nestin-knockdown reduces MERCs and capacity for differentiation in SLCs.

(a) qPCR analysis of relative mRNA expression of Nestin in Nestin-knockdown primary SLCs from 2 months old mice. (n = 3 biological repeats for each group; All data are mean ± SD; One-way ANOVA).

(b-c) Western Blot analysis and quantification of Nestin protein expression of Nestin-knockdown primary SLCs from 2 months old mice. (n = 3 biological repeats for each group; All data are mean ± SD; One-way ANOVA).

(d) Representative immunostaining pictures of colocalization between mitochondria and ER in Nestin-knockdown primary SLCs from 2 months old mice. Mitochondria and ER are marked with Mitotracker and ER tracker, respectively. Scale bar, 10 μm.

(e-f) Quantification of the levels of colocalization in (F) (Manders' (of mitochondria) and Pearson's coefficients are shown for each condition, n = 57, 52 and 45 cells for Control, shNES-1 and shNES-2 group, respectively; All data are mean ± SD; One-way ANOVA).

(g) Flow cytometry of intracellular ROS level stained with DHE of Nestin-knockdown primary SLCs from 2 months old mice in vitro. 100 μM antimycin was the positive control added to the Control group.

(h) Quantification of mean fluorescence intensity of DHE in (I). (n = 3 biological repeats for each group; All data are mean ± SD; One-way ANOVA).

(i) Representative SA-β-Gal staining pictures of Nestin-knockdown primary SLCs from 2 months old mice. Scale bar, 75 μm.

(j) Quantification of percentage of SA-β-Gal positive cells in (D). (n = 3 biological repeats for each group; All data are mean ± SD; One-way ANOVA).

(k) qPCR analysis of relative mRNA expression of testosterone production related genes at day 9 during induced differentiation of Nestin-knockdown primary SLCs from 2 months old mice. (n = 3 biological repeats for each group; All data are mean ± SD; Two-way ANOVA).

(l) Representative immunostaining pictures of LCs induced from Nestin-knockdown primary SLCs from 2 months old mice at day 9. LCs are identified as HSD3β+/CYP11A1+/HSD17β3+ cells. Scale bar, 20 μm.

(m) Quantification of the mean fluorescent intensity in (L) (n = 3 biological repeats for each group; All data are mean ± SD; Multiple t tests).

(n) Quantification of testosterone level in the supernatants of medium during induced differentiation at different time points (day 3, day 6, day 9) of Nestin-knockdown primary SLCs from 2 months old mice (n = 3 biological repeats for each group; All data are mean ± SD; Two-way ANOVA).

Two-sided comparison; Error bars represent SDs. *p < 0.05, **p < 0.01, ***p < 0.001.

Uncropped western blots and source data are provided as a Source Data file.

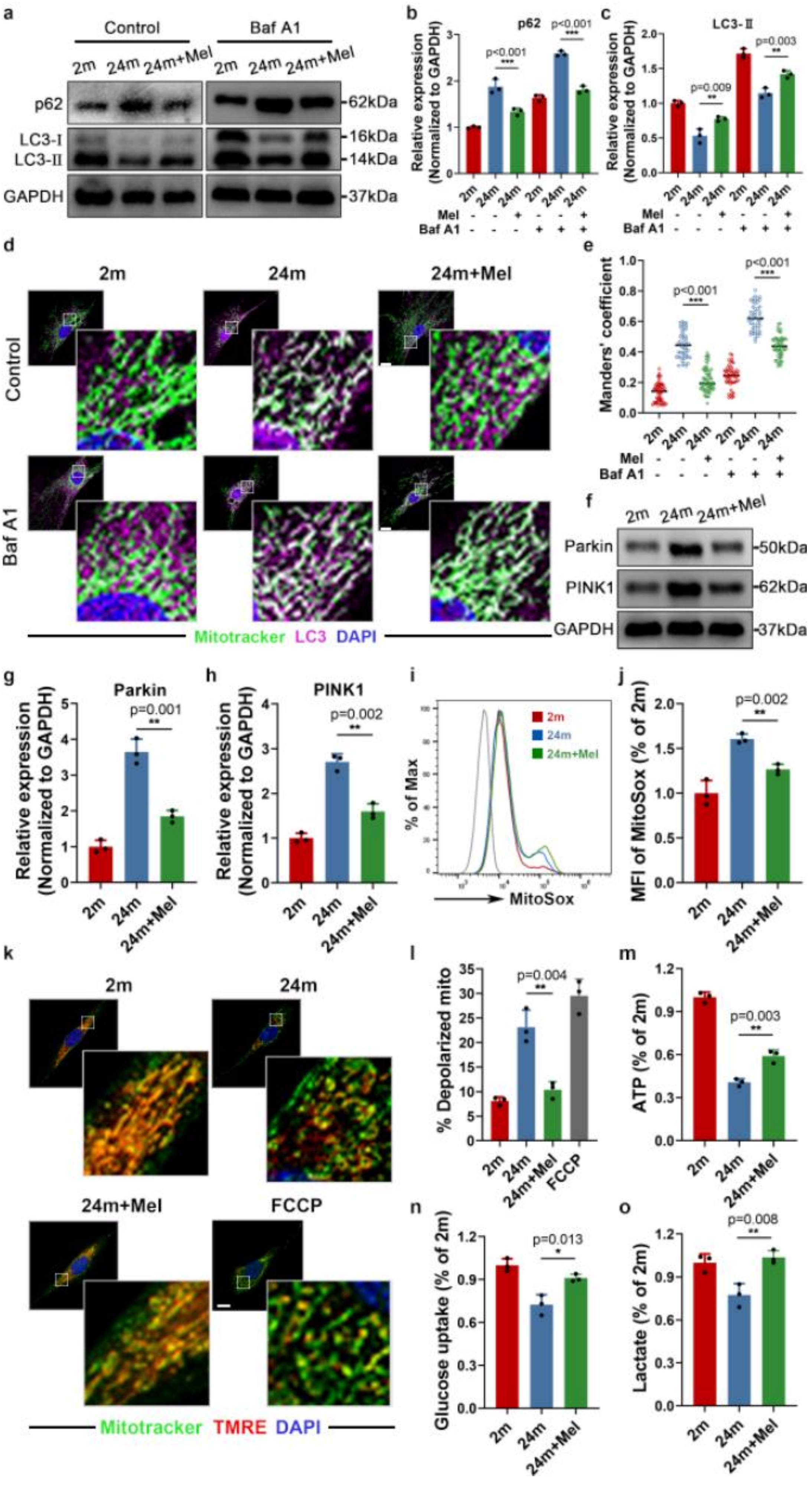
a
Control
Baf A1
2m 24m 24m+Mel 2m 24m 24m+Mel
p62
LC3-I
LC3-II
GAPDH
62kDa
16kDa
14kDa
37kDa
b
p62
Relative expression (Normalized to GAPDH)
p<0.001
p<0.001
Mel
Baf A1
c
LC3-II
p=0.009
p=0.003
d
2m
24m
24m+Mel
Control
Baf A1
Mitotracker LC3 DAPI
e
Manders' coefficient
p<0.001
p<0.001
f
Parkin
PINK1
GAPDH
50kDa
62kDa
37kDa
g
Parkin
p=0.001
h
PINK1
p=0.002
i
% of Max
MitoSox
2m
24m
24m+Mel
j
MFI of MitoSox (% of 2m)
p=0.002
k
2m
24m
24m+Mel
FCCP
Mitotracker TMRE DAPI
l
% Depolarized mito
p=0.004
m
ATP (% of 2m)
p=0.003
n
Glucose uptake (% of 2m)
p=0.013
o
Lactate (% of 2m)
p=0.008

Supplementary Fig. 8 | Melatonin prevents mitochondrial dysfunction resulting from SLC senescence.

(a-c) Western Blot analysis and quantification of autophagy related proteins in Control (Vehicle) and Bafilomycin A1 (Baf A1) treated primary Nestin-GFP+ SLCs from 2 months old, 24 months old and melatonin-treated 24 months old mice. (n = 3 biological repeats for each group; All data are mean ± SD; One-way ANOVA).

(d) Representative immunostaining pictures of mitophagy potential in Control (Vehicle) and Bafilomycin A1 (Baf A1) treated primary SLCs from 2 months old, 24 months old and melatonin-treated 24 months old mice. Mitochondria are stained with Mitotracker and autophagy is stained with LC3. Scale bar, 10 μm.

(e) Quantification of the level of colocalization in in (d). (Manders' coefficient of mitochondria is shown for each condition, n = 45, 44 and 50 cells for 2m, 24m and 24m+Mel group, respectively; All data are mean ± SD; One-way ANOVA).

(f-h) Western Blot analysis and quantification of mitophagy related proteins in primary SLCs from 2 months old, 24 months old and melatonin-treated 24 months old mice. (n = 3 biological repeats for each group; All data are mean ± SD; Unpaired t tests).

(i) Flow cytometry analysis of mitochondrial ROS in primary SLCs from 2 months old, 24 months old and melatonin-treated 24 months old mice.

(j) Quantitative analysis of mean fluorescence intensity of MitoSox staining in (f). (n = 3 biological repeats for each group; All data are mean ± SD; Unpaired t test).

(k) Representative immunostaining pictures of mitochondrial membrane potential stained with TMRE in primary SLCs from 2 months old, 24 months old and melatonin-treated 24 months old mice. Mitochondria are stained with Mitotracker. Scale bar, 10 μm. 20 μM FCCP was the positive control added to the 2m group.

(l) Quantification of percentage of depolarized mitochondria in (h). (n = 3 biological repeats for each group; All data are mean ± SD; Unpaired t test).

(m) Quantification of mitochondrial ATP production in primary SLCs from 2 months old, 24 months old and melatonin-treated 24 months old mice. (n = 3 biological repeats for each group; All data are mean ± SD; Unpaired t test).

(n) Quantification of glucose uptake in primary SLCs from 2 months old, 24 months old and melatonin-treated 24 months old mice. (n = 3 biological repeats for each group; All data are mean ± SD; Unpaired t test).

(o) Quantification of lactate production in primary SLCs from 2 months old, 24 months old and melatonin-treated 24 months old mice. (n = 3 biological repeats for each group; All data are mean ± SD; Unpaired t test).

Two-sided comparison; Error bars represent SDs. *p < 0.05, **p < 0.01, ***p < 0.001.

Uncropped western blots and source data are provided as a Source Data file.

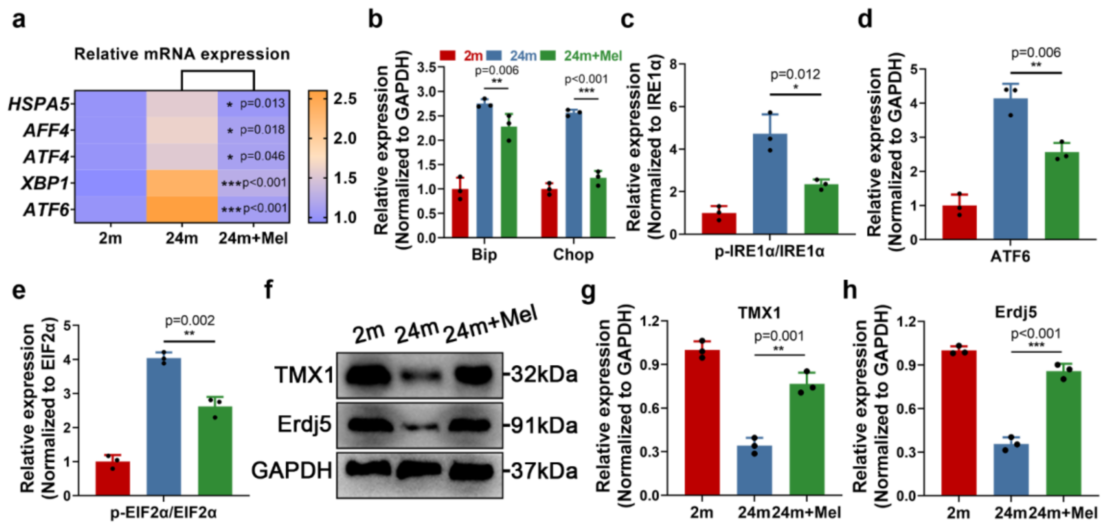

Supplementary Fig. 9 | Melatonin relieves ER stress resulting from SLC senescence.

(a) qPCR analysis of relative mRNA expression of ER stress genes in primary SLCs from 2 months old, 24 months old and melatonin-treated 24 months old mice. (n = 3 biological repeats for each group; All data are mean ± SD; Multiple t tests).

(b-e) Quantification of ER stress-related protein expression in primary SLCs from 2 months old, 24 months old and melatonin-treated 24 months old mice in **Figure 5f**. (n = 3 biological repeats for each group; All data are mean ± SD; b, multiple t test; d-e, unpaired t test).

(f-h) Western Blot analysis and quantification of the thioredoxin family proteins expression in primary SLCs from 2 months old, 24 months old and melatonin-treated 24 months old mice. (n = 3 biological repeats for each group; All data are mean ± SD; Unpaired t tests).

Two-sided comparison; Error bars represent SDs. *p < 0.05, **p < 0.01, ***p < 0.001.

Uncropped western blots and source data are provided as a Source Data file.

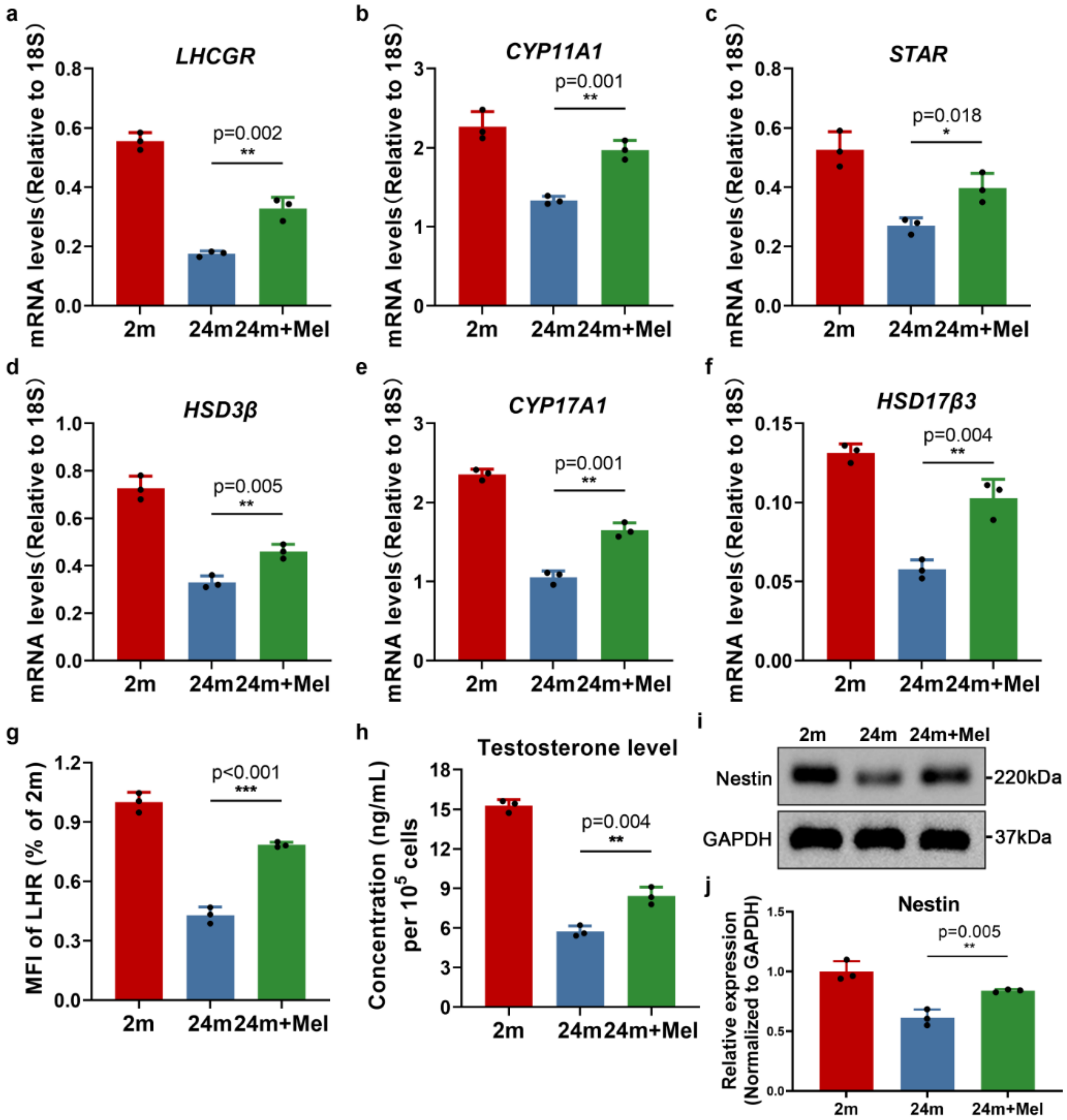

Supplementary Fig. 10 | Melatonin improves differentiation of aged SLCs in vitro and ex vivo.

(a-f) qPCR analysis of relative mRNA expression of testosterone production related genes in LCs induced by primary SLCs from 2 months old, 24 months old and melatonin-treated 24 months old mice at day 9. (n = 3 biological repeats for each group; All data are mean ± SD; Unpaired t test).

(g) Quantitative analysis of mean fluorescence intensity of LHR staining in **Figure. 5g**. (n = 3 biological repeats for each group; All data are mean ± SD; Unpaired t test).

(h) Quantitative analysis of testosterone level in the serum from 2 months old, 24 months old and melatonin-treated 24 months old mice in day 9. (n = 3 biological repeats for each group; All data are mean ± SD; Unpaired t test).

(i-j) Western Blot analysis and quantification of Nestin expression in seminiferous tubules from 2 months old, 24 months old and melatonin-treated 24 months old mice after treatment with melatonin for 5 days. (n = 3 biological repeats for each group; All data are mean ± SD; Unpaired t test).

Two-sided comparison; Error bars represent SDs. *$p < 0.05$, **$p < 0.01$, ***$p < 0.001$.

Uncropped western blots and source data are provided as a Source Data file.

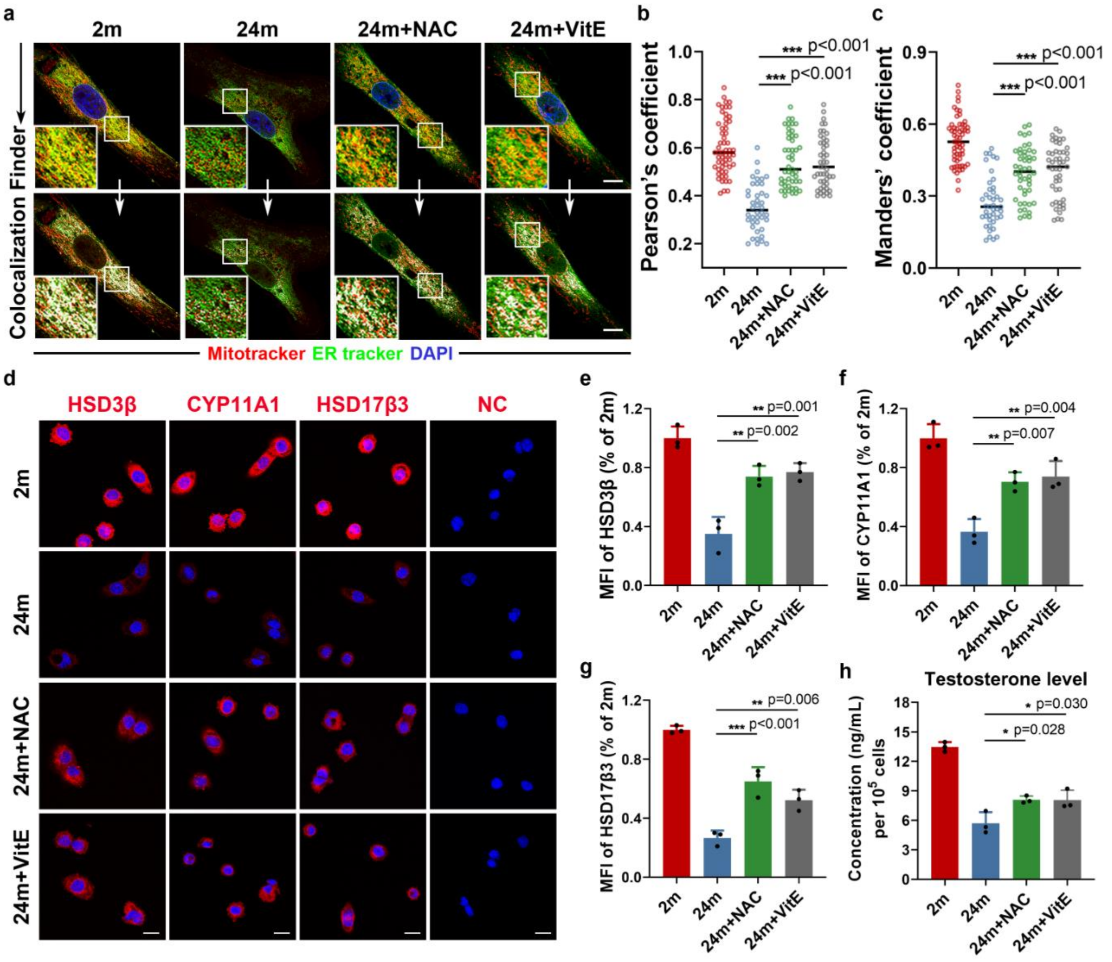

Supplementary Fig. 11 | Other antioxidants improves MERCs and capacity for differentiation in aged SLCs.

(a) Representative immunostaining pictures of colocalization between mitochondria and ER in primary SLCs from 2m, 24m, 24m+NAC and 24m+VitE treated group. Mitochondria and ER are marked with Mitotracker and ER tracker, respectively. Scale bar, 10 μm.

(b-c) Quantification of the levels of colocalization in (a) (Manders' (of mitochondria) and Pearson's coefficients are shown for each condition, n = 57, 42, 47 and 47 cells for 2m,24m, 24m+NAC and 24m+VitE group, respectively; All data are mean ± SD; One-way ANOVA).

(d) Representative immunostaining pictures of primary SLCs from 2m, 24m, 24m+NAC and 24m+VitE treated group after being induced to differentiation into LCs in day 9 in vitro. LCs are identified as HSD3β+/CYP11A1+/HSD17β3+ cells. Scale bar, 20 μm.

(e-g) Quantitative analysis of mean fluorescence intensity LC markers in (d). (n = 3 biological repeats for each group; All data are mean ± SD; One-way ANOVA).

(h) Measurement of testosterone concentration in the medium of primary SLCs from 2m, 24m, 24m+NAC and 24m+VitE treated group after being induced to differentiation into LCs in day 9 in vitro. (n = 3 biological repeats for each group; All data are mean ± SD; One-way ANOVA).

Two-sided comparison; Error bars represent SDs. *$p < 0.05$, **$p < 0.01$, ***$p < 0.001$.

Source data are provided as a Source Data file.

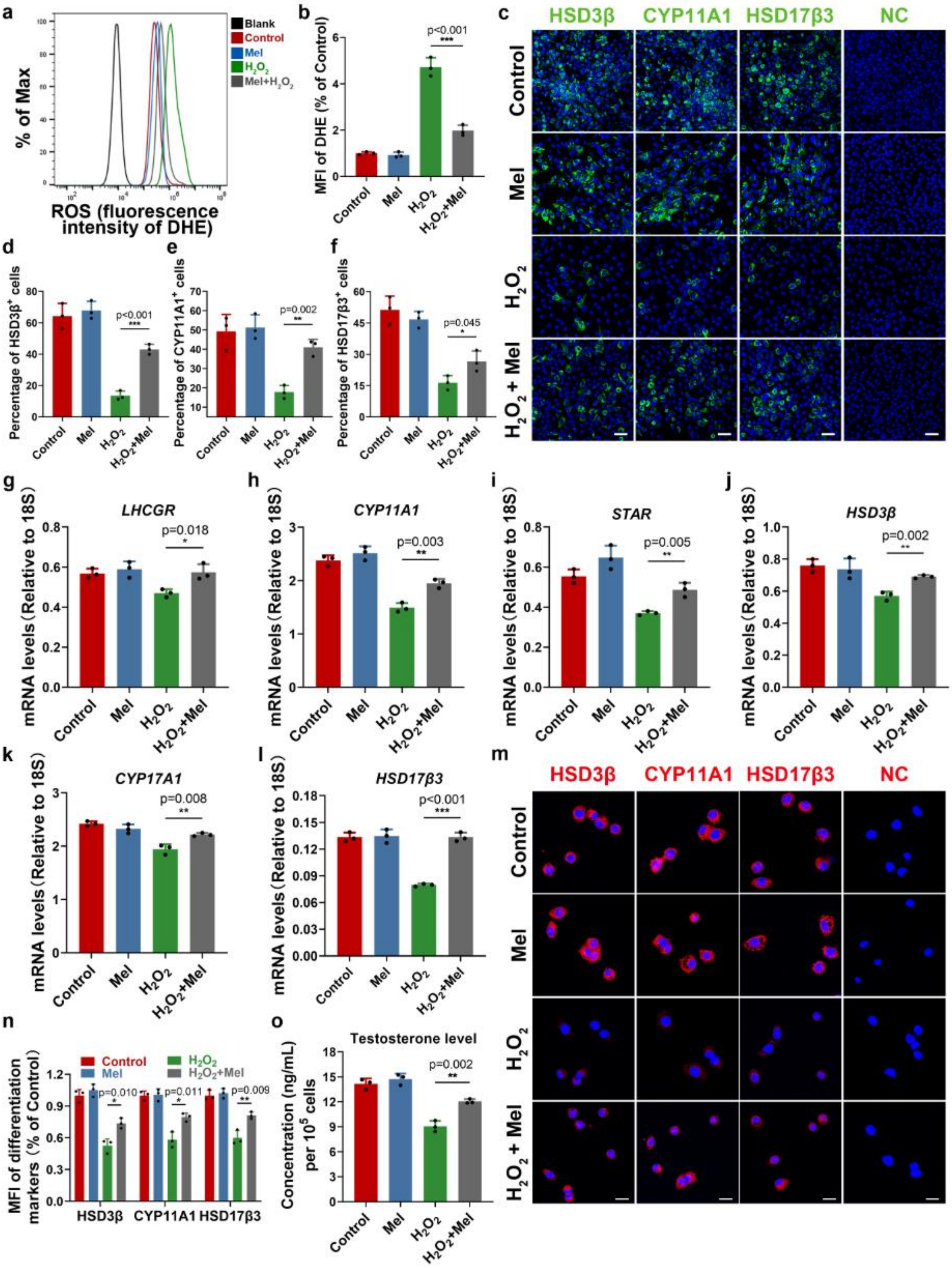

Supplementary Fig. 12 | Melatonin improves differentiation of young SLCs under oxidative stress conditions in vitro.

(a) Flow cytometry analysis of intracellular ROS in young SLCs under control, melatonin-treated, H_2O_2-treated and H_2O_2-melatonin combined treated from 7 days old mice. Intracellular ROS was stained with DHE.

(b) Quantitative analysis of mean fluorescence intensity of DHE in (a). (n = 3 biological repeats for each group; All data are mean ± SD; Unpaired t test).

(c) Representative immunostaining pictures of young SLCs under control, melatonin-treated, H_2O_2-treated and H_2O_2-melatonin combined treated from 7 days old mice after being induced to differentiation into LCs in day 9 in vitro. LCs are identified as HSD3β+/CYP11A1+/HSD17β3+ cells. Scale bar, 40 μm.

(d-f) Quantitative analysis of percentage of LCs with different markers of total cells in (c). (n = 3 biological repeats for each group; All data are mean ± SD; Unpaired t test).

(g-l) qPCR analysis of relative mRNA expression of testosterone production related genes in Nestin-GFP+ SLCs under control, melatonin-treated, H2O2-treated and H2O2-melatonin combined treated from 2 months old mice after being induced to differentiation into LCs in day 9 in vitro. (n = 3 biological repeats for each group; All data are mean ± SD; Unpaired t test).

(m) Representative immunostaining pictures of Nestin-GFP+ SLCs under control, melatonin-treated, H2O2-treated and H2O2-melatonin combined treated from 2 months old mice after being induced to differentiation into LCs in day 9 in vitro. LCs are identified as HSD3β+/CYP11A1+/HSD17β3+ cells. Scale bar, 20 μm.

(n) Quantitative analysis of mean fluorescence intensity LC markers in (G). (n = 3 biological repeats for each group; All data are mean ± SD; Multiple t tests).

(o) Measurement of testosterone concentration in the medium of Nestin-GFP+ SLCs under control, melatonin-treated, H_2O_2-treated and H_2O_2-melatonin combined treated from 2 months old mice after being induced to differentiation into LCs in day 9 in vitro. (n = 3 biological repeats for each group; All data are mean ± SD; Unpaired t test).

Two-sided comparison; Error bars represent SDs. *$p < 0.05$, **$p < 0.01$, ***$p < 0.001$.

Source data are provided as a Source Data file.

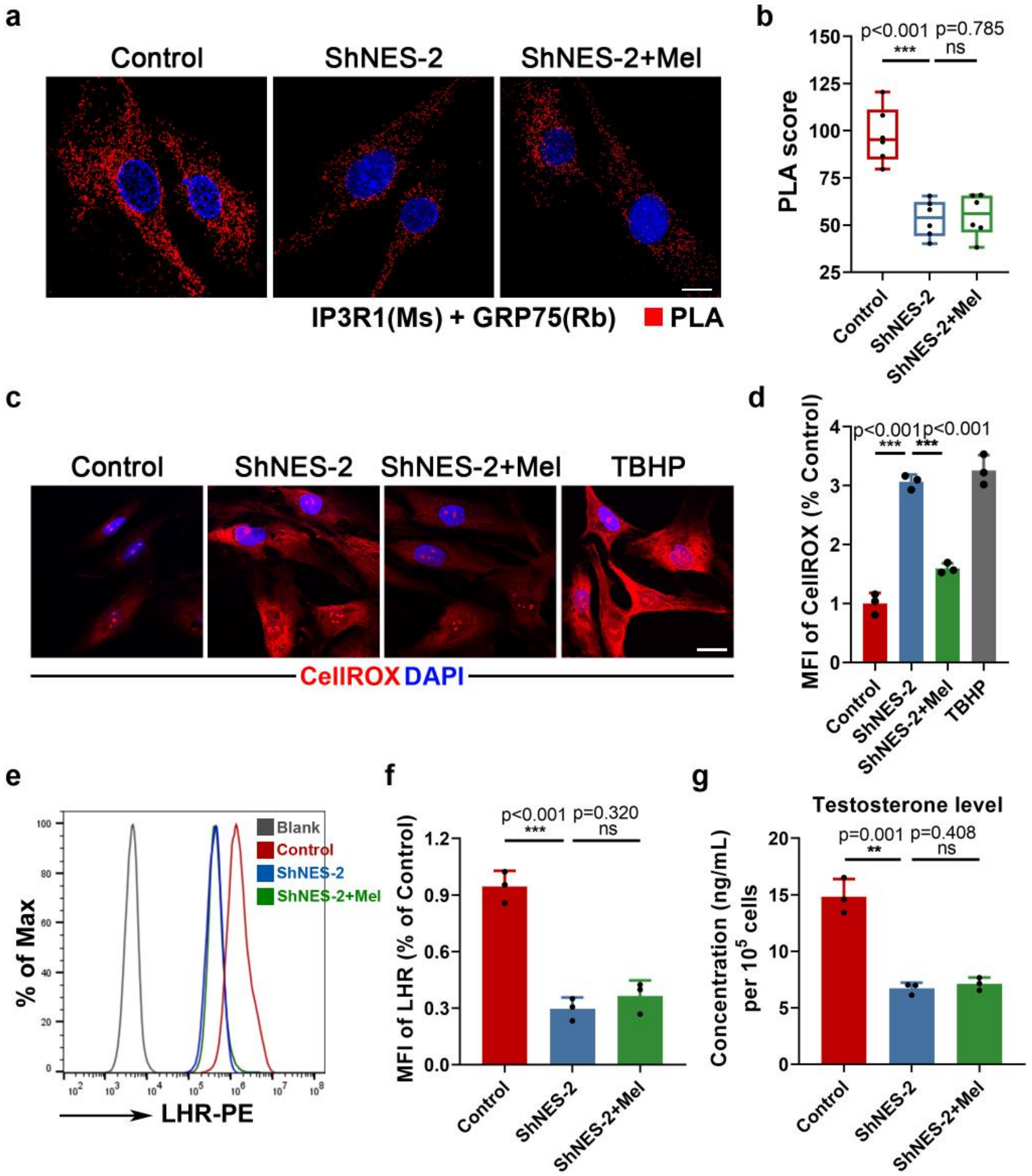

Supplementary Fig. 13 | Melatonin fails to rescue MERCs and capacity for differentiation in SLCs after Nestin knockdown.

(a) Representative PLA staining pictures of colocalization between mitochondria and ER in primary SLCs of Control, ShNES-2, ShNES-2 combined with melatonin group from 2 months old mice in vitro. Scale bar, 10 μm.

(b) Analysis of PLA score in (a) (n = 6 biological repeats for each group; Box plots indicate the median [center line inside the box], lower and upper

quartiles [bounds of box], smallest and largest values [whiskers], Unpaired t test).

(c) Representative immunostaining pictures of intracellular ROS stained with CellRox in primary SLCs of Control, ShNES-2, ShNES-2 combined with melatonin group from 2 months old mice in vitro. Scale bar, 20 μm. 200 μM TBHP was the positive control added to the Control group.

(d) Quantitative analysis of mean fluorescence intensity of CellRox in (c). (n = 3 biological repeats for each group; All data are mean ± SD; Unpaired t test).

(e) Flow cytometry analysis of LHR expression in LCs induced in primary SLCs of Control, ShNES-2, ShNES-2 combined with melatonin group from 2 months old mice in day 9.

(f) Quantitative analysis of mean fluorescence intensity of LHR staining in (e) (n = 3 biological repeats for each group; All data are mean ± SD; Unpaired t test).

(g) Quantification of testosterone level in the supernatants of medium culturing primary SLCs of Control, ShNES-2, ShNES-2 combined with melatonin group from 2 months old mice in vitro. (n = 3 biological repeats for each group; All data are mean ± SD; Two-way ANOVA).

(h) Quantitative analysis of testosterone level in the serum in primary SLCs of Control, ShNES-2, ShNES-2 combined with melatonin group from 2 months old mice in day 9. (n = 3 biological repeats for each group; All data are mean ± SD; Unpaired t test).

Two-sided comparison; Error bars represent SDs. $**p < 0.01$, $***p < 0.001$, ns, $p > 0.05$;

Source data are provided as a Source Data file.

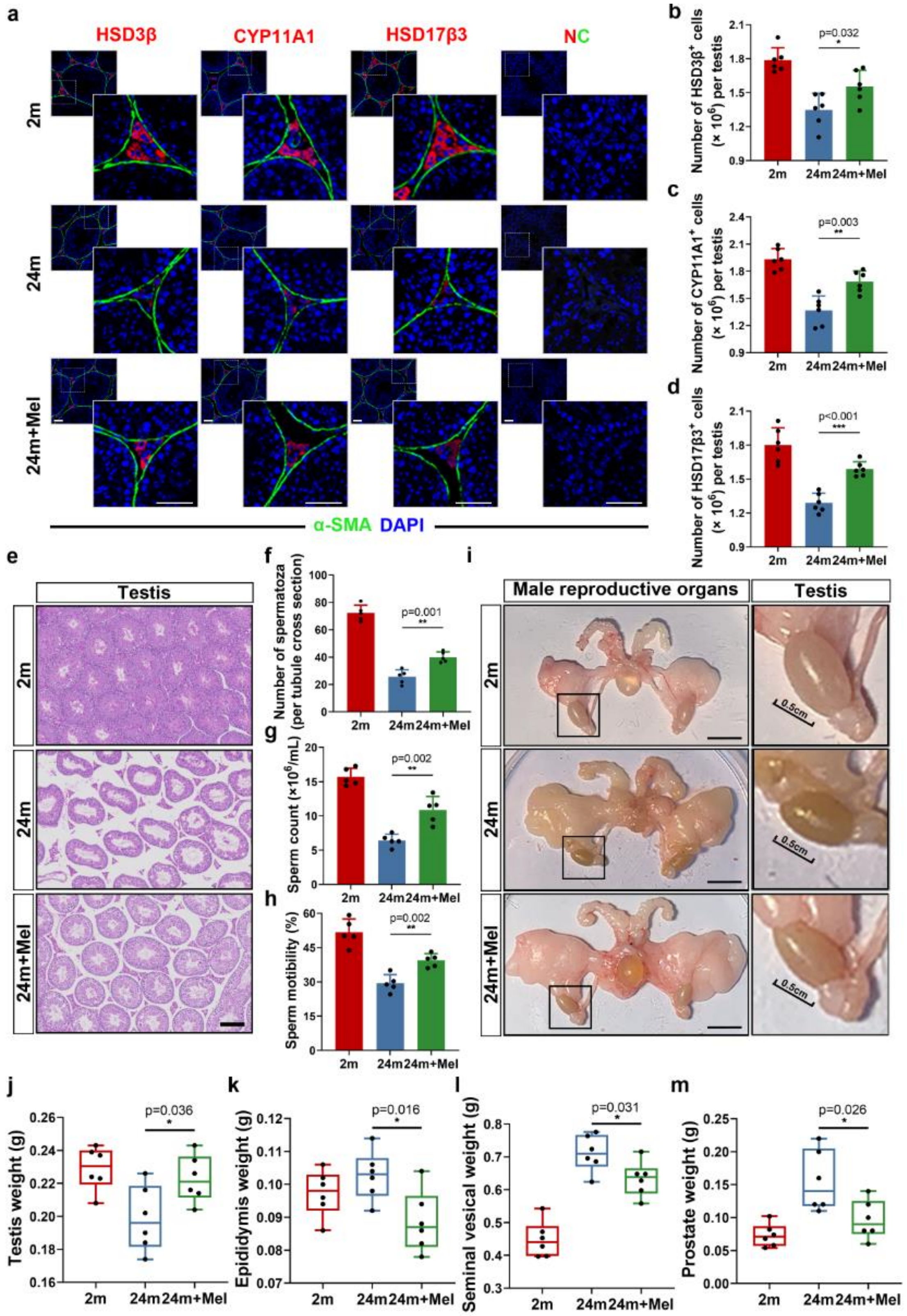

Supplementary Fig. 14 | Treatment with Melatonin increases testosterone level and promotes spermatogenesis in old mice.

(a) Representative immunostaining pictures of testicular mesenchyme from 2 months old, 24 months old and melatonin-treated 24 months old mice. LCs are identified as HSD3β+/CYP11A1+/HSD17β3+ cells. Scale bar, 40 μm for original pictures and 40 μm for enlarged pictures.

(b-d) Quantification of LCs cell numbers with different markers in (f). (n = 6 biological repeats for each group; All data are mean ± SD; Unpaired t test).

(e) Representative H&E staining pictures of testis from 2 months old, 24 months old and melatonin-treated 24 months old mice. Scale bar, 100 μm.

(f-h) Quantification of number of spermatozoa per seminiferous tubule cross section in testes, (f) sperm count of cauda epididymidis (g) and percentage of sperm motibility of cauda epididymidis (h) from 2 months old, 24 months old and melatonin-treated 24 months old mice. (n = 5 biological repeats for each group; All data are mean ± SD; Unpaired t test).

(i) General image of the whole reproductive system of 2 months old, 24 months old and melatonin-treated 24 months old mice. Scale bar, 1 cm for original pictures and 0.5 cm for enlarged pictures.

(j-m) Quantification of the change in weight of reproductive organs such as testis (j), epididymis (k), seminal vesicle glands (l) and prostate glands (m). (n = 6 biological repeats for each group; Box plots indicate the median [center line inside the box], lower and upper quartiles [bounds of box], smallest and largest values [whiskers]; Unpaired t test).

Two-sided comparison; Error bars represent SDs. *$p < 0.05$, **$p < 0.01$;

Source data are provided as a Source Data file.

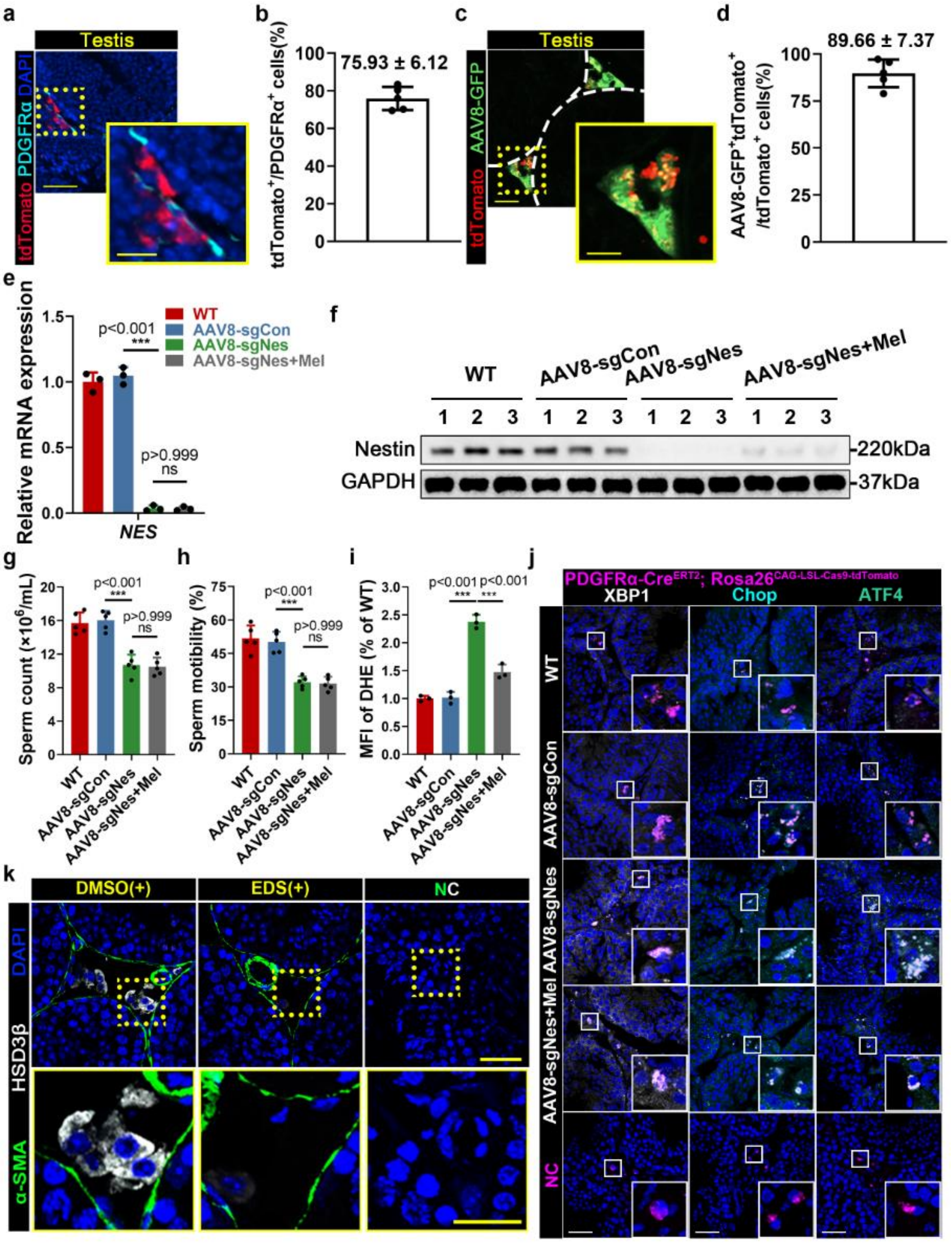

Supplementary Fig. 15 | AAV-mediated downregulation of Nestin separates MERCs and diminishes Melatonin's effect in attenuating male reproductive ageing.

(a) Representative immunostaining pictures of testicular mesenchyme from 2 months old PDGFRα-Cre^{ERT2}; Rosa26$^{CAG\text{-}LSL\text{-}Cas9\text{-}tdTomato}$ mice induced by

Tamoxifen i.p. and SLCs are identified as PDGFRα+ cells. Scale bar, 50 μm for original pictures and 20 μm for enlarged pictures.

(b) Quantification of percentage of PDGFRα+ cells in tdTomato+ cells in (a). (n = 5 biological repeats for each group; All data are mean ± SD).

(c) Immunofluorescence analysis was carried out to determine the co-localization of AAV8-GFP (green) and PDGFRα (red). 30 μm for original pictures and 15 μm for enlarged pictures.

(d) Statistical analysis of efficacy of AAV8 transduction in PDGFRα+ SLCs (n = 5 biological repeats for each group; All data are mean ± SD).

(e) qPCR analysis of relative mRNA expression of Nestin. (n = 3 biological repeats for each group; All data are mean ± SD; One-way ANOVA and Sidak's multiple comparisons test)

(f) Western Blot analysis of Nestin expression in the testis of wild type, AAV8-sgCon, AAV8-sgNes and AAV8-sgNes+Mel groups.

(g-h) Quantification of sperm count and sperm motibility of cauda epididymidis from wild type, AAV8-sgCon, AAV8-sgNes and AAV8-sgNes+Mel groups. (n = 5 biological repeats for each group; All data are mean ± SD; One-way ANOVA and Sidak's multiple comparisons test).

(i) Quantification of mean fluorescence intensity of DHE in **Figure. 7i**. (n = 3 biological repeats for each group; All data are mean ± SD; One-way ANOVA and Sidak's multiple comparisons test).

(j) Representative immunostaining pictures of testicular mesenchyme from wild type, AAV8-sgCon, AAV8-sgNes and AAV8-sgNes+Mel groups. ER stress-related proteins are identified as XBP1, Chop and ATF4. Scale bar, 40 μm.

(k) Representative immunostaining pictures of testicular mesenchyme from DMSO(+) and EDS(+) groups after 4 days treatment with EDS. LCs are identified as HSD3β+ cells. Scale bar, 40 μm for original pictures and 20 μm for enlarged pictures.

Two-sided comparison; Error bars represent SDs. *$p < 0.05$, ***$p < 0.001$, ns, $p > 0.05$;

Uncropped western blots and source data are provided as a Source Data file.

Supplementary Table 1 | Quantification for serum testosterone level of 2 months old male mice after 4 days of EDS treatment in different groups.

EDS(+) Mouse No.	Serum testosterone (ng/mL)	EDS(+)/Mel(+) Mouse No.	Serum testosterone (ng/mL)	EDS(+)/Mel(+) /AAV8-sgCon(+) Mouse No.	Serum testosterone (ng/mL)	EDS(+)/Mel(+) /AAV8-sgNes(+) Mouse No.	Serum testosterone (ng/mL)
**#1**	1.54	**#1**	0.35	**#1**	1.21	**#1**	0.98
**#2**	0.48	**#2**	4.29	**#2**	0.99	**#2**	0.31
**#3**	2.35	**#3**	1.48	**#3**	0.55	**#3**	1.45
**#4**	1.24	**#4**	0.99	**#4**	2.14	**#4**	2.21
**#5**	2.30	**#5**	2.88	**#5**	2.64	**#5**	0.11
**#6**	0.96	**#6**	1.16	**#6**	1.45	**#6**	1.18
**#7**	2.30	**#7**	4.18	**#7**	1.32	**#7**	5.02
**#8**	2.35	**#8**	3.8	**#8**	0.24	**#8**	2.42
**#9**	1.24	**#9**	4.44	**#9**	1.15	**#9**	2.46
**#10**	2.48	**#10**	0.69	**#10**	2.98	**#10**	0.65
**#11**	0.25	**#11**	1.72	**#11**	2.01	**#11**	5.16
**#12**	3.54	**#12**	1.91	**#12**	3.75	**#12**	1.3
**#13**	5.11	**#13**	4.06	**#13**	4.9	**#13**	3.92
**#14**	2.31	**#14**	5.13	**#14**	3.82	**#14**	2.75
**#15**	1.21	**#15**	0.74	**#15**	0.42	**#15**	4.43
**#16**	0.69	**#16**	4.08	**#16**	0.91	**#16**	4.78
**#17**	2.01	**#17**	0.78	**#17**	1.09	**#17**	1.51
**#18**	1.4	**#18**	4.32	**#18**	2.71	**#18**	0.59
**#19**	1.06	**#19**	0.91	**#19**	3.99	**#19**	2.75
**#20**	2.37	**#20**	2.18	**#20**	0.38	**#20**	3.37
**#21**	6.24	**#21**	2.93	**#21**	5.12	**#21**	1.78
**#22**	7.25	**#22**	2.68	**#22**	1.41	**#22**	5.16
**#23**	0.81	**#23**	0.42	**#23**	4.51	**#23**	0.47
**#24**	2.17	**#24**	1.24	**#24**	5.12	**#24**	1.14
**#25**	5.86	**#25**	2.54	**#25**	3.5	**#25**	1.56
**#26**	4.18	**#26**	0.38	**#26**	1.54	**#26**	0.52
**#27**	4.51	**#27**	1.26	**#27**	0.65	**#27**	2.14
**#28**	2.41	**#28**	1.98	**#28**	5.48	**#28**	1.45
**#29**	0.44	**#29**	5.24	**#29**	1.47	**#29**	1.32
**#30**	3.32	**#30**	7.32	**#30**	4.04	**#30**	1.22

EDS: ethylene dimethane sulfonate; Mel: melatonin.

Supplementary Table 2 | Key resource table.

REAGENT or RESOURCE	SOURCE	IDENTIFIER
Primary Antibodies		
WB：		
Anti-Nestin (1:200)	Santa cruz biotechnology	Cat#: sc-23927
Anti-Nrf2 (1:1000)	Abcam	Cat#: ab89443
Anti-GCLM (1:1000)	Proteintech	Cat#: 14241-1-AP
Anti-HO-1 (1:1000)	Cell Signaling Technology	Cat#: 5853
Anti-NQO1 (1:1000)	Abcam	Cat#: ab34173
Anti-Bip (1:1000)	Cell Signaling Technology	Cat#: 3177
Anti-Chop (1:1000)	Cell Signaling Technology	Cat#: 2895
Anti-p-IRE1α (1:1000)	Abcam	Cat#: ab48187
Anti-IRE1α (1:1000)	Cell Signaling Technology	Cat#: 3294
Anti-ATF6 (1:1000)	Cell Signaling Technology	Cat#: 65880
Anti-p-EIF2α (1:1000)	Cell Signaling Technology	Cat#: 3597
Anti-EIF2α (1:1000)	Cell Signaling Technology	Cat#: 9722
Anti-GAPDH (1:1000)	Cell Signaling Technology	Cat#: 2118S
Anti-LC3 A/B (1:1000)	Cell Signaling Technology	Cat#: 12741
Anti-Actin (1:1000)	Abcam	Cat#: ab8226
Anti-α-Tubulin (1:1000)	Cell Signaling Technology	Cat#: 3873
Anti-Keap1 (1:1000)	Cell Signaling Technology	Cat#: 8047
Anti-cMyc (1:1000)	Cell Signaling Technology	Cat#: 2278
Anti-Ubqutin (1:200)	Santa cruz biotechnology	Cat#: sc-8017
Anti-INF2 (1:1000)	SAB	Cat#: 39559
Anti-p16-ARC (1:100)	Santa cruz biotechnology	Cat#: sc-166760
Anti-p34-ARC (1:100)	Santa cruz biotechnology	Cat#: sc-515754
Anti-TMX1 (1:1000)	Sigma-Aldrich	Cat#: SAB1305470
Anti-DnaJC10 (1:100)	Santa cruz biotechnology	Cat#: sc-514624
Anti-IP3R-II (1:100)	Santa cruz biotechnology	Cat#: sc-398434
Anti-MFN2 (1:100)	Santa cruz biotechnology	Cat#: sc-515647
Anti-PACS2 (1:500)	Proteintech	Cat#: 19508-1-AP
Anti-GRP75 (1:100)	Santa cruz biotechnology	Cat#: sc-133137
Anti-Sig1R (1:500)	Abcam	Cat#: ab53852
Anti-p62 (1:1000)	Cell Signaling Technology	Cat#: 5114
Anti-Parkin (1:200)	Santa cruz biotechnology	Cat#: sc-32282
Anti-PINK1 (1:200)	Santa cruz biotechnology	Cat#: sc-517353
Anti-TXN1 (1:100)	Santa cruz biotechnology	Cat#: sc-271281
Anti-TrxR1 (1:100)	Santa cruz biotechnology	Cat#: sc-28321
Anti-Tom20 (1:200)	Santa cruz biotechnology	Cat#: sc-11415
Anti-Tim23 (1:100)	Santa cruz biotechnology	Cat#: sc-13298
ICC：		
Anti-Nestin (1:100)	Millipore	Cat#: Mab353
Anti-Nestin (1:200)	Novus	Cat#: NBP1-02419
Anti-α-SMA (1:100)	Arigo(arigo)	Cat#: ARG52485
Anti-HSD3B2 (1:100)	GeneTex	Cat#: GTX102744
Anti-CYP11A1 (1:100)	GeneTex	Cat#: GTX56293
Anti-HSD17B3 (1:100)	GeneTex	Cat#: GTX114480
Anti-Tom20 (1:200)	Santa cruz biotechnology	Cat#: sc-11415
Anti-α-Tubulin (1:1000)	Cell Signaling Technology	Cat#: 3873
Anti-SYCP-3 (1:200)	Santa cruz biotechnology	Cat#: sc-74569
Anti-Sox9 (1:200)	Millipore	Cat#: ab5535
Anti-XBP1 (1:50)	Abcam	Cat#: ab37152

Anti-Chop (1:400)	Cell Signaling Technology	Cat#: 2895
Anti-ATF6 (1:100)	Abcam	Cat#: ab37149
Anti-VDAC1/Porin (1:200)	Abcam	Cat#: ab15895
Anti-IP3R1 (1:50)	Santa cruz biotechnology	Cat#: sc-377518
PLA：		
Anti-GPR75 (1:100)	Cell Signaling Technology	Cat#: 3593
Anti-IP3R1 (1:50)	Santa cruz biotechnology	Cat#: sc-377518
IP：		
Anti-Keap1 (1:100)	Santa cruz biotechnology	Cat#: sc-15246
Anti-cMyc (1:250)	Cell Signaling Technology	Cat#: 2276s
Flow：		
Anti-LHR (1:100)	Abcam	Cat#: ab204950
Secondary Antibodies		
WB：		
Anti-mouse IgG, HRP-linked Antibody (1:5000)	Cell Signaling Technology	Cat#: 7076
Anti-rabbit IgG, HRP-linked Antibody (1:5000)	Cell Signaling Technology	Cat#: 7074
Anti-goat IgG HRP-linked Antibody (1:5000)	Abcam	Cat#: ab6885
ICC:		
Anti-mouse IgG conjugated to Alexa Fluor 488 (1:500)	Invitrogen	Cat#: A-11001
Anti-mouse IgG conjugated to Alexa Fluor 555 (1:500)	Invitrogen	Cat#: A-21422
Anti-rabbit IgG conjugated to Alexa Fluor 488 (1:500)	Invitrogen	Cat#: A-11008
Anti-rabbit IgG conjugated to Alexa Fluor 555 (1:500)	Invitrogen	Cat#: A-21428
Anti-mouse IgG conjugated to Alexa Fluor 647 (1:500)	Invitrogen	Cat#: A-21240
Chemicals, peptides, and recombinant proteins		
Triton X-100	Sigma-Aldrich	Cat#: T9284
Phalloidin-iFluro 555 Reagent	Abcam	Cat#: ab176756
TRIzol reagent	Molecular Research Center, Inc	Cat#: RT 111
Dihydroethidium	Sigma-Aldrich	Cat#: 37291
Cell ROX deep red reagent	ThermoFisher	Cat#: C10422
Tert-Butyl hydroperoxide solution (TBHP)	Sigma-Aldrich	Cat#: 416665
MItoSOX Red	ThermoFisher	Cat#: M36008
Tetramethylrhodamine ethyl ester perchlorate	Sigma-Aldrich	Cat#: 87917
FCCP	Sigma-Aldrich	Cat#: C2920
MitoTracker®Red CMXRos	Cell Signaling Technology	Cat#: 9082S
ER-Tracker™ Green	Cell Signaling Technology	Cat#: 8787S
MG132	Sigma-Aldrich	Cat#: M7449
Tamoxifen Citrate	Sigma-Aldrich	Cat#: 579000
Melatonin	Sigma-Aldrich	Cat#: M5250
EDS	(Li et al., 2016)	Renshan Ge (kind gift)
Bafilomycin A1	Sigma-Aldrich	Cat#: B1793
EGF	PeproTech	Cat#: AF-100-15
bFGF	Invitrogen	Cat#: 13256029
PDGF-BB	PeproTech	Cat#: 100-14B
Oncostatin M	PeproTech	Cat#: 300-10T
Dexamethasone	Sigma-Aldrich	Cat#: D1756
LIF	Millipore	Cat#: LIF1010
Insulin-transferrin-sodium selenite	Sigma-Aldrich	Cat#: 11074547001

Chick Embryo Extract	US Biologicals	Cat#: C3999
β-mercaptoethanol	Invitrogen	Cat#: 21985023
Non-Essential Amino Acids	HyClone	Cat#: SH30050.03
N2 supplement	Invitrogen	Cat#: 17502001
B27 supplement	Invitrogen	Cat#: A1486701
PDGF-AA	PeproTech	Cat#: 100-13A
LH	R&D Systems	Cat#: 8899-LH-010
Forskolin, Fsk	Sigma-Aldrich	Cat#: F6886
Smoothened Agonist HCl, SAG	Millipore	Cat#: 566660
IGF1	PeproTech	Cat#: 350-10
Collagenase, Type IV	Invitrogen	Cat#: 17104019
DMEM/F12	GIBCO	Cat#: 11320033
M199 medium	GIBCO	Cat#: C11150500BT
Bovine serum albumin	Invitrogen	Cat#: A34787
DMSO	Sigma-Aldrich	Cat#: D2605
Puromycin	Themofisher Scientific	Cat#: A1113803
Corn oil	Sigma-Aldrich	Cat#: 23-0230
FBS	GIBCO	Cat#: 10099
Critical commercial assays		
DHE (Dihydroethidium) Assay	Abcam	Cat#: ab236206
CellTiter-Glo® Luminescent Cell Viability Assay	Promega	Cat#: G7573
Lactate-Glo™ Assay	Promega	Cat#: J5022
Glucose Uptake-Glo™ Assay	Promega	Cat#: J1343
Testosterone Parameter Assay Kit	R&D Systems	Cat#: KGE010
Annexin V/propidium iodide (PI) assay kit	BIOSCI BIOTECH	Cat#: E606336
Duolink® In Situ Red Starter Kit Mouse/Rabbit	Sigma-Aldrich	Cat#: DUO92101
RevertAid First Strand cDNA Synthesis Kit	ThermoFisher	Cat#: K1622
FastStart Essential DNA Green Master Mix	Roche	Cat#: 06924204001
RNAiMAX Transfection Reagent	Invitrogen	Cat#: 13778030
BCA Protein Assay Kit	ThermoFisher	Cat#: 23227
UltraSensitiveTM SP (Mouse/Rabbit) IHC Kit	MXB	Cat#: KIT-9710
DAB Amplification Kit	MXB	Cat#: MAX-001
Senescence-associated β-galactosidase kit	Beyotime	Cat#: C0602
Glutathione GSH/GSSG Assay Kit	Sigma-Aldrich	Cat#: MAK440
Thioredoxin Reductase Kit	Sigma-Aldrich	Cat#: MAK409
Experimental models: Organisms/strains		
C57BL/6 mice	Beijing Vital River Laboratory	Cat#: C57-219-2
Nestin-GFP mice	(Yamaguchi et al., 2000)	Masahrio Yamaguchi (kind gift)
Rosa26^{RFP} mice	The Jackson Laboratory	JAX: 007914
PDGFRα-Cre^{ERT2} mice	(Marcelin et al., 2020; Tripathi et al., 2010)	Institute of Biochemistry and Cell Biology, Shanghai Institutes for Biological Sciences, Chinese Academy of Sciences (kind gift)
Nestin$^{loxP/loxP}$ mice	VIEWSOLID BIOTECH V	
Rosa26$^{CAG-LSL-Cas9-tdTomato}$ mice	GemPharmatech Co.,Ltd	Cat#: T002249
Oligonucleotides		
qPCR		
Forward: 5′-GCAGGAGAAGCAGGGTCTAC-3′<br>Reverse: 5′-GGGGTCAGGAAAGCCAA-3′	Sangon Biotech	mNESTIN

Forward: 5′-GCCTTCGAGACCCCAAGAAG-3′<br>Reverse: 5′-AAGGCTCCAGCTGGCATTAG-3′	Sangon Biotech	mH3D3β
Forward: 5′-CACTGAGACTCCACCCCATC-3′<br>Reverse: 5′-GGCAAAGCTAGCCACCTGTA-3′	Sangon Biotech	mCYP11A1
Forward: 5′-CAATGTTGTACTTATTAGTCGG-3′<br>Reverse: 5′-ATTTCTAAGCCTTCAAGGTGT-3′	Sangon Biotech	mHSD17β3
Forward: 5′-TTTGGCAACTTGACAGTCC-3′<br>Reverse: 5′-CTCCCTGTCTGCCAGTCT-3′	Sangon Biotech	mLHCGR
Forward: 5′-CCGGAGCAGAGTGGTGTCA-3′<br>Reverse: 5′-CAGTGGATGAAGCACCATGC-3′	Sangon Biotech	mSTAR
Forward: 5′-GGAGAGTTTGCCATCCCGAA-3′<br>Reverse: 5′-GAGCCTCTCCAATGCACGAT-3′	Sangon Biotech	mCYP17A1
Forward: 5′-GGACATGGAGCAAGTTTGGC-3′<br>Reverse: 5′-CCAGCGAGGAGATCGATGAG-3′	Sangon Biotech	mNFE2L2
Forward: 5′-GAATCGAGCAGAACCAGCCT-3′<br>Reverse: 5′-CTCAGCATTCTCGGCTTGGA-3′	Sangon Biotech	mHMOX1
Forward: 5′-CGCCTGAGCCCAGATATTGT-3′<br>Reverse: 5′-GCACTCTCTCAAACCAGCCT-3′	Sangon Biotech	mNQO1
Forward: 5′-AGTTGACATGGCATGCTCCG-3′<br>Reverse: 5′-TCCATCTTCAATCGGAGGCG-3′	Sangon Biotech	mGCLM
Forward: 5′-GACGGGCAAAGATGTCAGGA-3′<br>Reverse: 5′-GCCCGTTTGGCCTTTTCTAC-3′	Sangon Biotech	mBIP
Forward: 5′-AGAACCAGGAAACGGAAACAGA-3′<br>Reverse: 5′-TCTCCTTCATGCGCTGCTTT-3′	Sangon Biotech	mCHOP
Forward: 5′-GCTAAGGCGGGCTCCTCCGA-3′<br>Reverse: 5′-ACCCAACAGGGCATCCAAGTCG-3′	Sangon Biotech	mATF4
Forward: 5′-CTGAGTCCGAATCAGGTGCAG-3′<br>Reverse: 5′-ATCCATGGGGAGATGTTCTGG-3′	Sangon Biotech	msXBP1
Forward: 5′-AGTTGACATGGCATGCTCCG-3′<br>Reverse: 5′-TCCATCTTCAATCGGAGGCG-3′	Sangon Biotech	mATF6
Forward: 5′-GTAACCCGTTGAACCCCATT-3′<br>Reverse: 5′-CCATCCAATCGGTAGTAGCG-3′	Sangon Biotech	m18S
shRNA		
TRC clone ID: TRCN0000099447<br>Mouse Keap1 (50868)	Sigma-Aldrich	ShKeap1
5′-GGAAGAAGTTCCCAGGCTTCT-3′	Constructed in our lab	ShNES-1
5′-GCTGAAGCTGCATTTCCTTGG-3′	Constructed in our lab	ShNES-2
spgRNA (AAV)		
5′-GAGTCAGATCGCTCAGATCC-3′	Vigene Biosciences	Nestin<br>spgRNA（AAV-8）
5′-GTGTAGTTCGACCATTCGTG-3′	Vigene Biosciences	Scramble<br>spgRNA（AAV-8）
Software and algorithms		
ZEISS ZEN	ZEISS	https://www.zeiss.com.cn/microscopy/products/microscope-software/zen.html
GraphPad Prism	Graphpad	https://www.graphpad-prism.cn/
FlowJo	FlowJo	https://www.flowjo.com/
ImageJ	ImageJ	https://imagej.nih.gov/ij/
Imaris	Imaris	https://imaris.oxinst.com/
